# A Rapid Evaluation of the US Federal Tobacco 21 (T21) Law and Lessons From Statewide T21 Policies: Findings From Population-Level Surveys

**DOI:** 10.5888/pcd19.210430

**Published:** 2022-06-02

**Authors:** Israel T. Agaku, Lungile Nkosi, Queen D. Agaku, Joy Gwar, Tina Tsafa

**Affiliations:** 1 Harvard School of Dental Medicine, Boston, Massachusetts; 2 Sefako Makgatho Health Sciences University, Pretoria, South Africa; 3 Zatum LLC, Grand Blanc, Michigan; 4 Federal Medical Center, Makurdi, Benue, Nigeria; 5 Benue State University, Makurdi, Benue, Nigeria

## Abstract

**Background:**

On December 20, 2019, the minimum age for purchasing tobacco in the US was raised nationally to 21 years. We evaluated this law (Tobacco 21 [T21]) 1 year after implementation. We also compared states with versus without T21 policies during 2019 to explore potential equity impacts of T21 policies.

**Methods:**

We examined shifts in tobacco access among 6th through 12th graders using the National Youth Tobacco Survey. To explore equity of state T21 policies among youths and young adults, the associations with tobacco use were explored separately for race and ethnicity by using data from the 2019 Behavioral Risk Factor Surveillance System (for persons aged 18 to 20 years) and the 2019 Youth Risk Behavior Survey (for high school students).

**Results:**

The overall percentage of 6th to 12th graders perceiving that it was easy to buy tobacco products from a store decreased from 2019 (67.2%) to 2020 (58.9%). However, only 17.0% of students who attempted buying cigarettes in 2020 were unsuccessful because of their age. In the 2019 BRFSS, those aged 18 to 20 years living in a state with T21 policies had a lower likelihood of being a current cigarette smoker (adjusted prevalence ratio [APR], 0.58) or smoking cigarettes daily (APR, 0.41). Similar significant associations were seen when analyses were restricted to only non-Hispanic White participants but not for participants who were non-Hispanic Black, non-Hispanic Asian, Hispanic, or of other races or ethnicities. Consistent findings were seen among high school students.

**Conclusion:**

Greater compliance with the federal T21 law is needed as most youth who attempted buying cigarettes in 2020 were successful. Comparative analysis of states with versus states without statewide T21 policies in 2019 suggest the policies were differentially more protective of non-Hispanic White participants than other participants. Equitable and intensified enforcement of T21 policies can benefit public health.

**Summary**

**What is already known on this topic?**

On December 20, 2019, the minimum age for purchasing tobacco was raised nationally to 21 years.

**What is added by this report?**

One year after the federal law went into effect, the percentage of US middle and high school students who perceived it was easy to buy tobacco products from a store dropped by 8 percentage points, yet no change was seen for perceived ease of buying tobacco products online. Examination of state T21 policies that had existed before the federal T21 law suggested that the policies appeared to benefit non-Hispanic White students and young adults more than racial and ethnic minority students and young adults.

**What are the implications for public health practice?**

Comprehensive implementation of evidence-based tobacco use prevention and control programs through an equity lens can benefit public health.

## Introduction

Effective December 20, 2019, the US passed a federal law raising the minimum age of purchasing tobacco products from 18 years to 21 years (Tobacco 21 [T21]). This federal law was motivated by a large body of evidence showing that states and jurisdictions that had passed such policies subsequently witnessed reductions in tobacco consumption (1–5). The first 2 states to implement statewide T21 policies were Hawaii (January 1, 2016) and California (June 9, 2016) (6). New Jersey, Maine, and Oregon followed next in 2017 (7). Jurisdictions in various states also passed widely varying levels of T21 coverage of the population aged 18 to 20 years (8).

Evaluations of state and local T21 policies have been conducted by using sales and population-level data, both showing reduced overall tobacco consumption as an immediate effect (3,5). After California and Hawaii passed statewide T21 policies, monthly cigarette volume sales declined between January 2014 and December 2018 for both California (13.1%) and Hawaii (18.2%), compared with western states that had not implemented the T21 laws (6). Results from another study in Hawaii showed a significant drop in average monthly cigarette unit sales, together with a significant drop in menthol cigarette market share (9). During 2015 through 2019, cigarette sales declined for brands disproportionately used by smokers under the age of 21 (10). Friedman and Wu (11) assessed the effectiveness of local T21 policies across metropolitan and micropolitan statistical areas and metropolitan divisions (MMSAs); they found that the likelihood of smoking among youths living in an MMSA with full T21 coverage was reduced by 3.1% and among those living in an MMSA with partial T21 coverage, it was reduced by 1.2%.

To date, however, no study has evaluated the impact of the federal T21 law on tobacco use behaviors and perceptions. Furthermore, from an implementation perspective, it is important to glean insights learned from statewide implementation of T21 policies so they can be applied to the nationwide law. Our study was therefore conducted from both evaluation and implementation research perspectives. Our rapid evaluation sought to answer the question “One year after its passage, what has been the impact of the federal T21 law on tobacco use behaviors and perceptions among youths?” From an implementation research perspective, we sought to answer the following secondary questions: 1) Did the impact of state T21 policies on tobacco use behaviors vary among the different race and ethnic groups during 2019? (ie, was there effect modification by race or ethnicity when comparing tobacco-related outcomes between states with and states without T21 policies within strata of race or ethnicity?); and 2) Was there a differential effect of state T21 policies on cigarettes versus noncigarette tobacco products? Noncigarette tobacco products assessed were smokeless tobacco, e-cigarettes, and cigars. Because social contacts are important for tobacco access among youths who are generally price-sensitive (12), and because high-school–aged youths (especially those aged 16 and 17) may be socially aligned with 18-year-olds, we also sought to compare how the impact of state T21 policies compared between the primary target of T21 policies (persons aged 18–20 years) and high school youths.

## Methods

### Data sources

Within the secondary data analysis, multiple publicly available de-identified data sets were explored to answer the research questions. All analyzed data sets drew probabilistic samples from the noninstitutionalized US population. These data sets were the 2011 through 2020 National Youth Tobacco Survey (NYTS) — an annual school-based survey of US middle and high school students; the 2019 Youth Risk Behavior Survey (YRBS) — a biennial school-based survey of US high school students; and the 2019 and 2020 waves of the Behavioral Risk Factor Surveillance System (BRFSS) — an annual telephone-based survey of US adults aged 18 years or older (from which we restricted analyses to the population of young adults aged 18 to 20 years). YRBS yields both national and state-specific estimates; BRFSS yields only state-specific estimates, and NYTS yields only national estimates. With T21 now being a federal law, NYTS was used to explore shifts in perceived ease of access of tobacco products and tobacco purchase among youths. YRBS and BRFSS were used to explore lessons from statewide T21 policies, comparing implementation outcomes between those living in states with and states without T21 policies during 2019. Data were collected from these data sources on various indicators, including tobacco use, access patterns for different tobacco products, and sociodemographic characteristics.

With the passing of time and the collection of more years of postimplementation data, longer-term evaluation of the federal T21 policy would be important to guide public health programs, policy, and practice. Our rapid evaluation, while conducted 1 year after policy implementation, is still valid because the data are fit for use and fit for purpose. It is inevitable that a large segment of the population surveyed in 2020 was potentially exposed to the federal T21 policy even if there was a time lag between enactment and enforcement. For example, over half (61.3%) of the 2020 BRFSS participants were surveyed between June 2020 and January 2021, suggesting that most of the 2020 survey population were potentially exposed to the federal T21 law even if we assumed that the law had no measurable influence until mid-2020 or thereafter.

### Measures

#### Evaluation outcomes for the federal T21 Law

With the first full year of the federal T21 law (ie, 2020) coinciding with the first full year of COVID-19, evaluating the effect of the federal T21 law on tobacco consumption is challenging, considering evidence showing COVID-19–attributable increase in tobacco consumption (13,14). Consequently, our evaluation framework considered not only consumption but also attitudes and perceptions specific to tobacco access among youths.

In NYTS, perceived ease of minors buying tobacco products from a store and online was defined as a response of “easy” or “somewhat easy” (vs “not easy at all”) to the question “How easy do you think it is for people your age to buy tobacco products [in a store/online]?” Students were further asked, “During the past 30 days, did anyone ever refuse to sell you cigarettes because of your age?” Categorical response options were: “I did not try to buy cigarettes in a store during the past 30 days,” “No, no one refused because of my age,” and “Yes, someone refused because of my age.” Either of the latter 2 responses was classified as having made an attempt in the past 30 days to buy cigarettes. A response of “No, no one refused because of my age” was classified as having made a successful cigarette purchase.

#### Implementation outcomes from statewide T21 policies

With half a decade having passed since the first statewide T21 policies in California and Hawaii, we were interested in implementation-related end points addressing the equity impact of T21 policies. Secondary research questions were 1) What equity impact did state T21 policies have along the lines of race or ethnicity? and 2) Was there a differential effect of state T21 policies on cigarettes vs noncigarette tobacco products? Our interest in examining whether the effect of statewide T21 policies was different among those who were White, versus those of racial or ethnic minority groups, was motivated by previous research showing more lax enforcement of access laws in communities of color coupled with targeted marketing of tobacco products in those same communities by the tobacco industry (2,15–17). Addressing any potential uneven impact of T21 policies across tobacco product types has equity relevance, considering the popularity of some noncigarette products among certain subgroups (eg, cigars among Black respondents [18,19]). Noncigarette tobacco products assessed in our study included smokeless tobacco, e-cigarettes, and cigars (current use defined as past 30-day use in YRBS or as use every day or on some days in BRFSS where applicable). For cigarettes, we explored outcomes that discriminated between frequent and nonfrequent smoking, based on findings from studies showing reduced consumption following implementation of statewide T21 policies (3,5,11). In BRFSS, we examined the following 3 outcomes representing increasingly higher frequency of cigarette smoking: smoked at least 100 cigarettes regardless of whether they now smoke (ie, cumulative threshold of cigarettes smoked); current cigarette smoker (ie, smoked at least 100 cigarettes and smoke every day or some days); and daily cigarette smoker (ie, smoked at least 100 cigarettes and smoke every day). In YRBS, we examined the following 2 outcomes: heavy cigarette smoking (ie, smoke ≥11 cigarettes per day [CPD]) and any past 30-day smoking (smoked ≥1 of the past 30 days).

In the 2019 cycles of YRBS and BRFSS, we classified states as having statewide T21 policies if said policies had been enacted and had gone into effect about a year before the passage of the federal T21 law on December 20, 2019 (California, Hawaii, Maine, Massachusetts, New Jersey, and Oregon [20]). Conversely, states that had never enacted statewide T21 policies, or those whose enacted T21 policies were not set to go into effect until 2019 or thereafter, were classified as not having T21 statewide policies as of the study period (all other states).

### Analysis

The percentage of US middle and high school students participating in NYTS who attempted buying cigarettes from a store in the past 30 days, and the percentage who were denied a cigarette sale because of their age among those who tried, was assessed overall across survey years and further stratified by school level, sex, and racial and ethnic group (Hispanic, non-Hispanic White, non-Hispanic Black, non-Hispanic Asian, and non-Hispanic other race [American Indian/Alaska Natives, Native Hawaiian/Other Pacific Islander, and multiracial]). Exploratory logistic regression analysis was used to explore correlates of perceived ease of buying tobacco products from a store and of making a successful cigarette purchase from a store among all students during 2020. Among those aged 18 to 20 years participating in the BRFSS in each state, we computed the percentage who reported current and daily cigarette smoking and compared between 2019 and 2020 using χ^2^ tests (significant at *P* < .05).

To measure the equity impact of statewide T21 policies as our implementation-related outcomes, we contrasted tobacco use outcomes (current cigarette, cigar, smokeless tobacco, and e-cigarette use) between those living in states with and states without statewide T21 policies in 2019, overall and within strata of race and ethnicity. Adjusted prevalence ratios (APRs) were calculated, adjusting for sociodemographic factors and alcohol use. The year 2019 was used because it preceded the federal T21 policy, allowing a distinction to be made between states exposed to T21 versus those unexposed. The year 2019 also preceded the COVID-19 pandemic in the US, thus eliminating potential confounding by the pandemic. All analyses were weighted and performed with Stata version 14 (StataCorp LLC).

## Results

### Changes in tobacco use and perceptions

Within BRFSS, only 4 states had a significant decline in prevalence of current cigarette smoking among those aged 18 to 20 years during 2019 and 2020: Delaware, Florida, North Carolina, and West Virginia. In contrast, 8 states saw a decline in prevalence of current daily cigarette smoking among those aged 18 to 20 years during 2019 and 2020: Delaware, Maine, Nebraska, Nevada, New Mexico, Oklahoma, Pennsylvania, and South Dakota. During 2020, a total of 311,361 young adults aged 18 to 20 years smoked cigarettes daily, based on weighted population counts from all 50 states and the District of Columbia. The states with the highest prevalence of current daily cigarette smoking among those aged 18 to 20 years were Kentucky (7.2%), New Mexico (7.2%), Oregon (7.2%), Oklahoma (7.3%), Alaska (7.6%), Arkansas (8.9%), North Dakota (10.8%), Montana (11.7%), Mississippi (12.4%), Wyoming (14.4%), and Tennessee (18.1%).

Analysis of NYTS data revealed that the overall percentage of US middle and high school students who perceived it was easy to buy tobacco products from a store decreased overall between 2019 (67.2%) and 2020 (58.9%) (*P* < .001, Table 1). However, this shift in perception was significant only for perceived in-store access, not perceived online access. The overall percentage who perceived it was easy getting tobacco products online was high and did not change significantly between 2019 (86.6%) and 2020 (85.8%). Furthermore, in 2020, 76.0% of US middle and high school students who perceived it would be difficult getting tobacco products from a store felt it was easy getting them online. Although perceived ease of accessing tobacco products from a store increased with increasing grade level during 2020 (*P* trend < .001), no significant trend was observed for perceived ease accessing tobacco products online (*P* trend = .26) (Figure). Among racial or ethnic population subgroups, Black students were the only ones to report no significant change in perceived access of tobacco products from a store during 2011 through 2020 (Table 1).

**Table 1:** Percentage of US Middle and High School Students Who Perceived it Would Be Easy to Get Tobacco Products From a Store, National Youth Tobacco Survey, 2011–2020^a^

Characteristic	2011 (n = 18,866)	2012 (n = 24,658)	2013 (n = 18,406)	2014 (n = 22,007)	2016 (n = 20,675)	2017 (n = 17,872)	2018 (n = 20,189)	2019 (n = 19,018)	2020 (n = 14,531)
**Overall**	62.5 (61.5–63.5)	66.5 (65.7–67.2)	61.2 (60.3–62.1)	64.7 (63.9–65.6)	64.6 (63.7–65.4)	66.1 (65.2–67.1)	69.8 (69.0–70.6)	67.2 (66.4–68.0)	58.9 (57.9–59.9)
**Race and ethnicity**									
Black, non-Hispanic	57.5 (55.2–59.7)	62.8 (60.5–65.0)	56.6 (54.3–58.9)	63.4 (61.4–65.5)	59.3 (57.1–61.5)	62.6 (60.1–65.1)	64.7 (62.2–67.1)	67.2 (64.8–69.6)	57.4 (54.4–60.5)
Hispanic	62.7 (60.9–64.5)	65.7 (64.1–67.3)	62.0 (60.2–63.7)	62.9 (61.3–64.5)	64.6 (63.0–66.2)	67.0 (65.2–68.7)	67.8 (66.3–69.3)	67.1 (65.6–68.6)	59.9 (58.0–61.8)
Other, non-Hispanic^b^	60.3 (57.2–63.4)	67.4 (65.3–69.6)	59.0 (56.4–61.6)	66.5 (63.8–69.2)	64.7 (62.2–67.1)	67.2 (64.3–70.1)	71.5 (69.0–74.0)	68.5 (66.1–70.9)	59.4 (56.5–62.2)
White, non-Hispanic	64.6 (63.1–66.1)	68.1 (67.1–69.2)	63.7 (62.4–65.0)	66.2 (65.0–67.5)	66.9 (65.6–68.1)	67.4 (66.0–68.8)	72.6 (71.4–73.8)	67.5 (66.3–68.7)	58.9 (57.4–60.3)
**Sex**									
Male	63.8 (62.4–65.2)	64.8 (63.7–65.9)	63.0 (61.7–64.2)	62.0 (60.8–63.2)	62.1 (60.9–63.3)	64.1 (62.8–65.5)	66.8 (65.6–68.0)	64.1 (62.9–65.3)	56.5 (55.0–57.9)
Female	61.2 (59.7–62.6)	68.3 (67.2–69.3)	59.4 (58.1–60.7)	67.4 (66.2–68.6)	67.1 (65.9–68.3)	68.2 (66.9–69.5)	72.8 (71.7–74.0)	70.7 (69.5–71.8)	61.4 (60.0–62.8)
**School level**									
Middle (grades 6–8)	41.9 (40.2–43.5)	48.2 (46.9–49.4)	40.6 (39.2–42.0)	44.8 (43.4–46.2)	46.7 (45.3–48.1)	48.1 (46.6–49.6)	54.8 (53.5–56.2)	55.3 (54.0–56.6)	46.4 (44.9–47.8)
High (grades 9–12)	77.6 (76.5–78.6)	80.5 (79.7–81.3)	76.7 (75.7–77.8)	80.1 (79.2–81.0)	78.6 (77.7–79.6)	79.9 (78.9–80.9)	81.4 (80.5–82.3)	76.6 (75.6–77.6)	68.7 (67.4–70.0)
**Attempted cigarette purchase in past 30 days**									
No	60.1 (59.0–61.2)	65.7 (64.9–66.5)	59.1 (58.2–60.1)	64.1 (63.1–65.0)	64.6 (63.7–65.6)	66.1 (65.1–67.1)	70.1 (69.2–71.0)	67.3 (66.4–68.1)	58.8 (57.7–59.8)
Yes	83.8 (81.7–85.9)	75.7 (73.3–78.0)	83.8 (81.5–86.1)	69.9 (67.8–72.0)	65.0 (62.8–67.3)	67.4 (64.8–69.9)	68.2 (66.1–70.3)	66.4 (61.5–71.2)	59.6 (56.4–62.8)
**Age, y**									
9–12	32.3 (29.6–35.0)	36.7 (34.9–38.4)	30.3 (28.3–32.3)	34.1 (32.1–36.0)	38.9 (36.8–40.9)	37.6 (35.4–39.8)	44.5 (42.5–46.6)	48.5 (46.6–50.4)	39.7 (37.6–41.8)
13–15	58.3 (56.8–59.8)	65.4 (64.3–66.6)	57.2 (55.8–58.6)	62.4 (61.1–63.8)	62.1 (60.8–63.4)	66.2 (64.8–67.6)	69.3 (68.1–70.5)	66.8 (65.5–68.0)	58.7 (57.2–60.3)
16–18	81.9 (80.8–83.1)	83.4 (82.5–84.4)	81.4 (80.3–82.6)	82.9 (81.8–84.0)	81.4 (80.2–82.5)	81.8 (80.6–83.0)	83.6 (82.5–84.7)	77.8 (76.5–79.0)	70.9 (69.2–72.5)
≥19	73.2 (64.2–82.2)	78.2 (70.6–85.7)	82.5 (76.0–89.0)	79.6 (72.1–87.1)	83.3 (76.8–89.8)	83.2 (75.4–91.1)	79.0 (70.1–87.9)	70.2 (60.4–80.1)	68.5 (54.8–82.2)

a In the 2015 cycle of the survey, the question on perceived ease of buying tobacco from a store was not assessed. Data are shown as % (95% CI).

b Other races and ethnicities included in the survey were non-Hispanic American Indians/Alaska Natives, Native Hawaiians/other Pacific Islanders, and multiracial persons.

**Figure:**
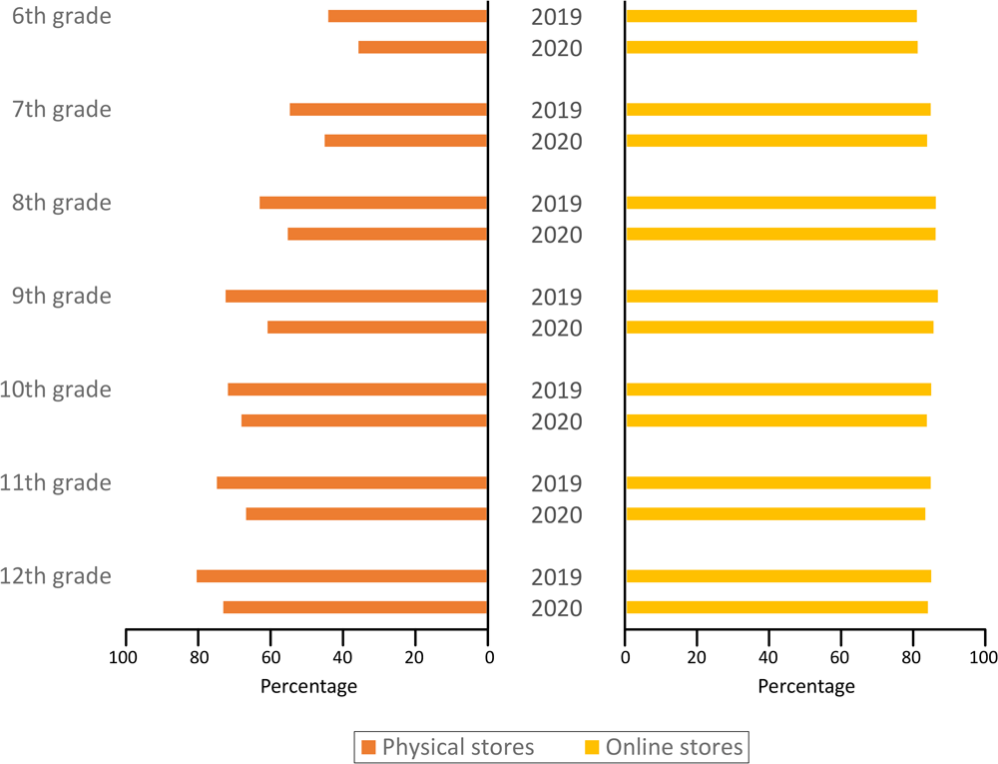
Changes by grade level between 2019 and 2020 in the percentage of students who perceived it would be easy to get tobacco products in a physical store as well as online, National Youth Tobacco Survey. Students were asked “How easy do you think it is for people your age to buy tobacco products in a store?” and “How easy do you think it is for people your age to buy tobacco products online?” Categorical response options were “easy,” “somewhat easy,” or “not easy at all.” Any response other than “not easy at all” was classified as perceiving buying tobacco products as easy.

**Table:** 

Grade	Perceived ease of buying tobacco from physical stores, %		Perceived ease of buying tobacco from online stores, %	
	2019	2020	2019	2020
6th	45.1	36.4	82.7	82.9
7th	56.0	46.1	86.6	85.6
8th	64.6	56.6	88.1	88.0
9th	74.3	62.4	88.7	87.5
10th	73.6	69.8	86.8	85.6
11th	76.8	68.5	86.7	85.1
12th	82.6	75.0	86.8	85.9

Overall, 10.1% of all US middle and high school students reported trying to buy a cigarette in the past 30 days, down from 14.1% in 2018 (*P* < .001). Attempted cigarette purchase in 2020 increased by grade level and was 2.7%, 4.3%, 5.4%, 9.6%, 15.3%, 16.0%, and 19.0% among students in the 6th, 7th, 8th, 9th, 10th, 11th, and 12th grades, respectively (*P* trend < .001). Most middle and high school students who tried purchasing cigarettes in the past 30 days successfully bought them, with only 17.0% overall reporting in 2020 that someone refused to sell cigarettes to them because they were underaged, an increase from 14.2% in 2018.

Multivariable logistic regression for all US middle and high school students during 2020 revealed that, whereas differences existed in the odds of perceiving that buying tobacco products from a store was easy along the lines of sex, race, and grade level, these differences were not significant when it came to reporting past 30-day success in purchasing cigarettes from a store among all students (Table 2). Perceived ease of buying tobacco products was lower among male than female students (adjusted odds ratio [AOR], 0.80; 95% CI, 0.74–0.88), higher among Hispanic students than non-Hispanic White students (AOR, 1.11; 95% CI, 1.01–1.23), and increased with increasing grade levels, being 1.54, 2.35, 2.99, 4.19, 3.95, and 5.46 among the 7th, 8th, 9th, 10th, 11th, and 12th grades, respectively, compared with the 6th grade (all *P* < .05). None of these factors, however, were associated with making a successful cigarette purchase in 2020.

**Table 2:** Factors Associated With Perceived Ease of Buying Tobacco Products From a Store and of Successfully Purchasing a Tobacco Product From a Store in the Past 30 Days, National Youth Tobacco Survey, 2020^a^

Characteristic	Perception that buying tobacco from a store is easy^b^		Successfully purchased cigarettes from a store in the past 30 days^c^	
	Adjusted odds ratio (95% CI)	*P* value	Adjusted odds ratio (95% CI)	*P* value
**Sex**				
Female	1 [Reference]		1 [Reference]	
Male	0.80 (0.74–0.88)	<.001	0.75 (0.53–1.07)	.11
**Race and ethnicity**				
Black, non-Hispanic	1.00 (0.87–1.16)	.99	1.05 (0.51–2.14)	.90
Hispanic	1.11 (1.01–1.23)	.047	0.76 (0.51–1.14)	.18
Other, non-Hispanic^d^	1.12 (0.97–1.28)	.12	1.19 (0.63–2.27)	.59
White, non-Hispanic	1 [Reference]		1 [Reference]	
**Grade level**				
6	1 [Reference]		1 [Reference]	
7	1.54 (1.32–1.79)	<.001	0.83 (0.29–2.41)	.74
8	2.35 (2.02–2.75)	<.001	1.35 (0.48–3.80)	.57
9	2.99 (2.54–3.51)	<.001	1.73 (0.63–4.76)	.29
10	4.19 (3.54–4.95)	<.001	1.40 (0.52–3.75)	.50
11	3.95 (3.34–4.67)	<.001	0.82 (0.31–2.14)	.68
12	5.46 (4.58–6.52)	<.001	0.60 (0.24–1.52)	.28

a Analysis adjusted for all factors listed in table.

b Students were asked “How easy do you think it is for people your age to buy tobacco products in a store?” Categorical response options were “easy,” “somewhat easy,” or “not easy at all.” Any response other than “not easy at all” was classified as perceiving buying tobacco products from a store was easy.

c Students were asked “During the past 30 days, did anyone ever refuse to sell you cigarettes because of your age?” Categorical response options were: “I did not try to buy cigarettes in a store during the past 30 days,” “No, no one refused because of my age,” and “Yes, someone refused because of my age.” A response of “No, no one refused because of my age” was classified as having made a successful cigarette purchase. All other responses were classified as having either made an unsuccessful attempt or no attempt at all.

d Other races and ethnicities included in the survey were non-Hispanic American Indians/Alaska Natives, Native Hawaiians/other Pacific Islanders, and multiracial persons.

### Differential effects of statewide T21 policies

Comparative analysis of states with versus without statewide T21 policies in 2019 revealed that such policies were differentially more protective of White adolescents than other adolescents and young adults. These findings were seen consistently in analyses of both the BRFSS data among those aged 18 to 20 years as well as within the YRBS among high school students. Results from BRFSS analysis of all those aged 18 to 20 years showed that those living in a state with T21 policies during 2019 had lower likelihood of reporting having smoked up to 100 cigarettes (APR, 0.71; 95% CI, 0.53–0.94), being a current cigarette smoker (APR, 0.58; 95% CI, 0.39–0.86), or smoking cigarettes daily (APR, 0.41; 95% CI, 0.23–0.74) (Table 3). Similar associations were seen when analyses were restricted to only White young adults aged 18 to 20 years; living in an area with statewide T21 policies was associated with lower likelihood of White young adults having smoked 100 cigarettes (APR, 0.69; 95% CI, 0.49–0.97), being current cigarette smokers (APR, 0.60; 95% CI, 0.39–0.92), or smoking cigarettes daily (APR, 0.34; 95% CI, 0.17–0.71). None of these associations were significant for young adults who were Black, Asian, Hispanic, or of other races or ethnicities. Conversely, among Black young adults, statewide T21 policies were associated with current smokeless tobacco use (APR, 11.98; 95% CI, 4.55–31.55).

**Table 3:** Associations Between Exposure to Statewide T21 Policies^a^ and Tobacco-Use–Related Outcomes Among Young Adults Aged 18–20 Years by Race and Ethnicity, Behavioral Risk Factor Surveillance System^b^, 2019^c^

Indicator	Adjusted prevalence ratio (95% CI)					
	Overall (n = 10,146)	Asian, non-Hispanic (n = 531)	Black, non-Hispanic (n = 809)	Hispanic (n = 1,955)	Other^d^ (n = 792)	White, non-Hispanic (n = 5,920)
Smoked up to 100 cigarettes	0.71 (0.53–0.94)	0.71 (0.26–1.96)	0.16 (0.02–1.19)	0.73 (0.42–1.27)	0.40 (0.19–0.85)	0.69 (0.49–0.97)
Smoke cigarettes daily	0.41 (0.23–0.74)	0.33 (0.07–1.62)	0.34 (0.04–3.08)	0.51 (0.13–1.94)	0.18 (0.04–0.79)	0.34 (0.17–0.71)
Current cigarette smokers (daily or nondaily)	0.58 (0.39–0.86)	0.49 (0.20–1.17)	0.23 (0.03–1.62)	0.47 (0.22–1.02)	0.43 (0.18–1.01)	0.60 (0.39–0.92)
Current smokeless tobacco users	1.21 (0.80–1.85)	0.39 (0.08–2.02)	11.98 (4.55–31.55)	0.97 (0.44–2.11)	0.55 (0.13–2.28)	1.24 (0.80–1.93)

^a^ Policies that prohibited the sale of tobacco products to anyone aged <21 years.

^b^ Race and ethnicity information were missing for 139 respondents.

^c^ Adjusted for binge drinking, sex, and income. Analyzed T21 states were California, Hawaii, Oregon, Maine, and Massachusetts. Data were not available for New Jersey as part of the 2019 survey. Data were available for the remaining 49 states and the District of Columbia. Analysis excluded US territories.

^d^ Other races and ethnicities included in the survey were non-Hispanic American Indians/Alaska Natives, Native Hawaiians/other Pacific Islanders, and multiracial persons.

Analysis of YRBS data on high school students showed consistent findings. Living in an area with statewide T21 laws during 2019 was associated with significantly lower likelihood of being a current smoker of cigars among all students combined (APR, 0.81; 95% CI, 0.70–0.94) as well as among the White subgroup (APR, 0.78; 95% CI, 0.67–0.92), but was not significant among students who were Black, Asian, Hispanic, or of other races or ethnicities (Table 4). Furthermore, state T21 policies were associated with reduced likelihood of heavy cigarette smoking among high school students (ie, smoking ≥11 cigarettes per day) for all racial or ethnic categories except Hispanic. State T21 policies were associated with reduced likelihood of any past 30-day cigarette smoking (ie, smoking ≥1 of the past 30 days) only among White and Hispanic high schoolers. Among White high schoolers who saw a significant association with state T21 policies for both heavy smoking (ie, ≥11 CPD) and any past 30-day smoking (smoking ≥1 of the past 30 days), the measure of association was stronger for the former. Specifically, living in a state with T21 policies reduced the probability of smoking 11 or more CPD among White high school students by 87% (APR, 0.13; 95% CI, 0.06–0.29), but reduced the probability of smoking on 1 or more of the past 30 days by only 33% (APR, 0.67; 95% CI, 0.47–0.97).

**Table 4:** Associations Between Exposure to Statewide T21 Policies^a^ and Tobacco-Use–Related Outcomes Among High School Students, by Race and Ethnicity, Youth Risk Behavior Survey^b^, 2019^c^

Indicator	Adjusted prevalence ratio (95% CI)					
	Overall (n = 182,491)	Asian, non-Hispanic (n = 8,547)	Black, non-Hispanic (n = 22,634)	Hispanic (n = 34,241)	Other^d^ (n = 16,026)	White, non-Hispanic (n = 95,945)
Current (past 30 days) cigar smoking	0.81 (0.70–0.94)	1.97 (0.81–4.79)	1.00 (0.60–1.65)	1.27 (0.84–1.94)	0.91 (0.63–1.31)	0.78 (0.67–0.92)
Current smokeless tobacco use	1.54 (0.77–3.12)	0.01 (0.00–0.03)	0.03 (0.01–0.11)	3.80 (1.87–7.69)	0.22 (0.04–1.10)	0.65 (0.26–1.64)
Current e-cigarette use	0.91 (0.79–1.04)	1.08 (0.74–1.57)	1.57 (0.97–2.54)	1.00 (0.81–1.24)	0.96 (0.73–1.26)	0.87 (0.75–1.01)
Current cigarette smoking (smoking ≥1 of the last 30 days)	0.70 (0.52–0.93)	1.51 (0.69–3.29)	1.50 (0.59–3.82)	0.39 (0.18–0.81)	0.79 (0.55–1.13)	0.67 (0.47–0.97)
Current heavy cigarette smoking (≥11 cigarettes per day)	0.62 (0.16–2.42)	0.01 (0.00–0.10)	0.04 (0.00–0.47)	0.92 (0.22–3.89)	0.01 (0.00–0.08)	0.13 (0.06–0.29)

^a^ Policies that prohibited the sale of tobacco products to anyone aged <21 years.

^b^ Race and ethnicity information were missing for 5,098 respondents.

^c^ Adjusted for grade, race, alcohol use, and sex. Analyzed T21 states were California, Hawaii, Maine, and New Jersey. Data were not available for Massachusetts because it was not one of the sampled states.

^d^ Other races and ethnicities included in the survey were non-Hispanic American Indians/Alaska Natives, Native Hawaiians/other Pacific Islanders, and multiracial persons.

Statewide T21 policies were not associated with e-cigarette use in the overall population, nor in any racial or ethnic stratum based on analysis of YRBS data. Statewide T21 policies were also not associated with smokeless tobacco use in the overall population; within race and ethnic subgroups, however, T21 policies were associated with increased likelihood of current smokeless tobacco use among Hispanic high schoolers (APR, 3.80; 95% CI, 1.87–7.69) but with lower likelihood of current use among Black and Asian high schoolers (Table 4).

## Discussion

Our findings suggest that the federal T21 law was not effectively enforced during 2020 because only 17.0% of US middle and high school students who attempted a cigarette purchase in that year reported that the salesclerk refused to sell it to them because they were underaged. Perceived ease of getting tobacco products from a store was higher among Hispanic students than among White participants. Furthermore, whereas every other racial or ethnic group had a significant decrease in the percentage who felt it would be easy to get tobacco products from a store during 2011 through 2020, this percentage did not change among Black participants (57.4% in 2020). Among those aged 18 to 20 years, we found that state T21 policies were protective against cigarette smoking among White young adults but not among those of racial or ethnic minority groups. These findings are consistent with previous research showing weaker age verification enforcements among minority populations (2,16,21). Several factors may account for these differences in tobacco access and use, including differences in accessibility to small retail stores where age verification is less likely to be enforced, such as liquor stores, gas stations, corner stores, small kiosks, and convenience stores (7,22,23). The tobacco industry has been well known to target tobacco products to neighborhoods with large Black populations (15,17,24). Reduction of illegal tobacco sales to minors through stronger enforcement of T21 can benefit public health.

Access laws in general may be best optimized when implemented in concert with other evidence-based tobacco use control and prevention measures because youths mostly access tobacco products from social contacts and could adopt adaptive behaviors such proxy purchasing (via an older adult), switching to less-regulated products, or buying from less-regulated or less-enforced environments (4,25–27). For example, while most social media sites have banned sales of tobacco products on their sites, illegal tobacco sellers have been known to evade such restrictions by misspelling brand names, using slang, or using expressions in foreign languages to communicate with potential customers (28). The basis for concern regarding these channels of tobacco access is that even young children may become very savvy with such online communication and tobacco access patterns. In our study, whereas students in high school were more confident than students in middle school about ease of tobacco access from a physical store, no differences existed by grade level in perceived ease of tobacco access online.

The strongest associations in our study for state T21 policies were observed for smoking intensity, suggesting that these policies may have a stronger impact in cutting down on smoking than quitting completely. For example, among White high school students, we found that state T21 policies reduced the probability of smoking 11 or more cigarettes per day by 87% but reduced the probability of smoking on 1 or more of the past 30 days by only 33%. Many previous studies have linked T21 policies with reduced volume sales of cigarettes (5,6,12). A pause or a reduction in tobacco use must be accompanied by an intention to stop smoking for it to qualify as a quit attempt (29). Therefore, interventions aimed at intrinsically motivating desire to quit among youths and young adults are needed.

### Limitations

This study has some limitations. First, all assessed measures were self-reported and may be subject to misreporting. Second, the cross-sectional data sets analyzed can only support associational inferences. Third, in our state-specific analysis, there may be misclassification of individuals covered by T21 policies because we based the classification on whether a statewide policy was in existence (substate geographic identifiers were unavailable in the datasets). Consequently, individuals from jurisdictions with T21 policies may have been classified as not being exposed to T21 policies if their entire state was not covered by such a policy. Finally, there may have been some confounding from the effect of COVID-19 that was not measured. However, the direction of the bias would be expected to be toward the null, considering varying levels of pandemic-related closure of schools and other social settings that would typically facilitate smoking behavior (such closures would conceivably restrict the social channels where youths might access tobacco products). Furthermore, with government-mandated shutdowns during parts of the COVID-19 pandemic (30), there may have been fewer opportunities for youths to attempt to buy tobacco products from stores compared with previous years. However, results from analysis that limited the analyzed data to only pre–COVID-19 or intra–COVID-19 years are less susceptible to potential bias in this regard because of restriction.

### Conclusion

Evaluation of the federal T21 law at the 1-year mark shows it has potential to reduce ease of tobacco access among adolescents and young adults, but intensified efforts are needed to increase compliance. Our results showed that over 4 in 5 US middle and high school students who attempted to buy cigarettes in the past 30 days during 2020 were successful because only 17.0% of those who attempted to do so reported that the salesclerk refused to sell to them because they were underaged. Efforts to increase tobacco retailers’ compliance to mandatory age checks are warranted in all communities, but especially among racial and ethnic minority communities for whom our findings suggest suboptimal enforcement of state access laws. The equitable and intensified enforcement of the federal T21 law among all racial and ethnic subgroups and across all tobacco products may achieve a positive equity impact in reducing all forms of tobacco use among US youths and young adults.
